# Postresuscitation pleth variability index-guided hemodynamic management of out-of-hospital cardiac arrest survivors: A randomised controlled trial

**DOI:** 10.1016/j.resplu.2025.100933

**Published:** 2025-03-19

**Authors:** Stefano Malinverni, Paul Dumay, Pierre Domont, Marc Claus, Antoine Herpain, Jolan Grignard, Silvia Matta, Fatima Zohra Bouazza, Queitan Ochogavia

**Affiliations:** a Emergency Department, Centre Hospitalier Universitaire Saint-Pierre, Université Libre de Bruxelles, Rue Haute 322, 1000 Brussels, Belgium; b Emergency Department, Hôpital de Nivelles, Centre Hospitalier Universitaire HELORA, Nivelles, Belgium; c Intensive Care Unit, Centre Hospitalier Universitaire Saint-Pierre, Université Libre de Bruxelles, Belgium

**Keywords:** Out-of-hospital cardiac arrest, Post-resuscitation care, Hemodynamic management, Lactate clearance, Lactate, Pleth variability index

## Abstract

**Background and purpose:**

Hypotension and shock after return of spontaneous circulation is harmful. Goal-directed post-resuscitation care aims at maintaining adequate perfusion pressure, but evidence.

on strategies to achieve this goal is limited. This study aimed to compare outcomes of pleth variability index (PVi) supported hemodynamic management during early hospital admission with those of standard hemodynamic management.

**Methods and trial design:**

From March 2019 to August 2023, all mechanically ventilated patients adults admitted alive after a non-traumatic out-of-hospital cardiac arrest (OHCA) to the emergency department of Saint-Pierre University Hospital in Brussels, were screened for inclusion in this prospective, parallel, randomised, single-blind study. We enrolled patients with signs of tissue hypoperfusion after cardiac arrest. Patients were randomly allocated (1:1) to undergo hemodynamic treatment based on the PVi (intervention) or standard monitoring (control). Hemodynamic interventions targeted mean blood pressure above 70 mmHg, a capillary refill time below 3 s and urine output above 0.5 ml/kg/minute. The primary outcome was lactate clearance at 3 h. We hypothesized that PVi guided hemodynamic management would result in a faster lactate clearance at 3 h.

**Results:**

96 patients underwent randomization. Due to non-consent and loss to follow-up 82 patients were included in the analysis, 39 in the intervention and 43 in the control group. The median lactate clearance 3 h after inclusion was not different between groups (57.4% [Interquartile range (IQR): 27.7–75.8%] in the control group versus 61.5% [IQR: 39.3–74.7%] in the intervention group), with a mean difference of 4.9% (95% CI, −7.5–17.2; *p* = 0.44) between the two groups. No side effects were observed.

**Conclusion:**

A pleth variability index-based protocol did not significantly improve the lactate clearance compared with standard care (NCT03841708).

## Introduction

The annual incidence of out-of-hospital cardiac arrest (OHCA) in Europe is 67 to 170 per 100,000 inhabitants and approximately 33% of patients achieve a return to spontaneous circulation (ROSC).

Following ROSC, shock and hypotension are frequent.^1^ Low mean arterial pressure (MAP) or diastolic pressure is associated with potential harm.^2^, ^3^ Although the primary hypoxic–ischaemic injury (HIBI) causes substantial brain damage, hypotension has been involved in a two-hit mechanism that results in neuronal death.^4^ Retrospective and prospective observational studies have reported that MAP above 65 mm Hg is associated with higher survival rates and better neurological outcomes^5^, ^6^ although prospective interventional trials have produced discordant results.^7^, ^8^

Goal-directed postresuscitation care aims to maintain adequate perfusion pressure for the brain but evidence is limited regarding specific blood-pressure targets and the methods required to achieve them.

Current guidelines provide few strong recommendations about post-OHCA care to avoid secondary brain tissue hypoxia and injury because of limited certainty of evidence.^9^, ^10^ The only strong recommendation is to maintain systolic blood pressure above 100 mmHg mainly through fluid infusion.^9^ However, hypotension may result from a combination of cardiogenic, vasoplegic and hypovolemic states, each with variable response to fluid administration. Fluid therapy does not always improve convective oxygen delivery, and in some cases may be harmful.^11^ In HIBI fluids can increase oedema which in turn will reduces oxygen diffusion to injured neuronal cells. The feasibility and safety of tailored and individualized fluid administration according to non-invasive indexes during the early phases of post-ROSC remain poorly studied.^10^ Randomised animal studies^13^ and observational studies^14^ have suggested a potential benefit of early individualized hemodynamic goal-directed therapy.

**Fig. 1:**
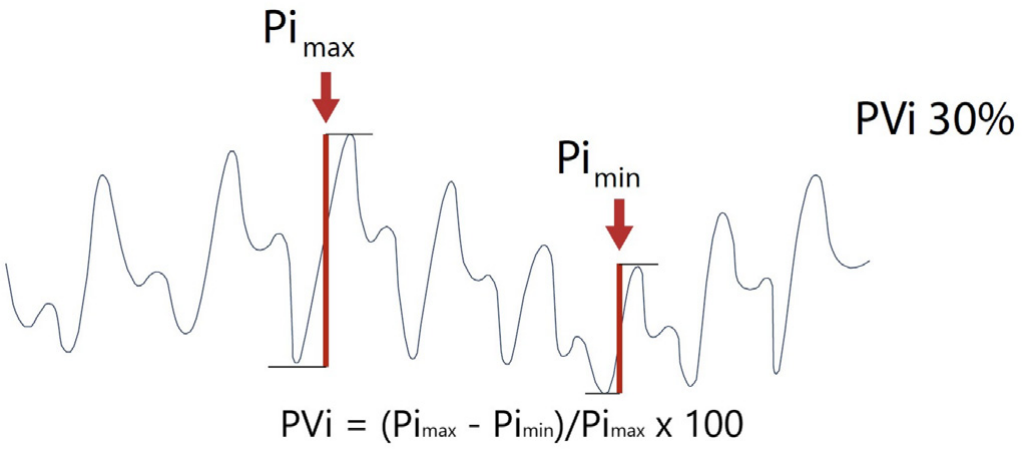
Physiologic principles and mechanism supporting the pleth variability index (PVi). Fig. 1 illustrates the physiological principles supporting the functioning of the pleth variability index (PVi). The intensity of the plethysmographic signal derived from a pulse oximeter, measured as pulsatility index, varies in accordance with stroke volume variations. In patients ventilated without asynchronies, variations in the PI measured at the finger tip are representative of heath lung interactions. PVi represents the ratio of (PI max − PI min)/PI max. Values above 13% are associated with fluid responsiveness.

The pleth variability index (PVi) is a dynamic index ranging between 0 and 100. It measures the relative respiratory variations in pulse oximetry plethysmographic waveform amplitude measured non-invasively from a pulse oximetry sensor (Fig. 1). During mechanical ventilation, in a well sedated or paralysed patient, PVi continuously assesses fluid responsiveness^15^ with values > 14% being associated with fluid responsiveness.^16^ PVi has the potential to guide initial resuscitation by informing relative fluid status and fluid responsiveness^17^, reduce excessive positive fluid balance by avoiding fluid therapy in unresponsive patients^18^ and support therapeutic hemodynamic decisions in the absence of invasive monitoring tools.^19^

We hypothesised that in-hospital hemodynamic management based on PVi and standard non-invasive monitoring would improve lactate clearance, and demonstrate a superior safety profile in terms of fluid balance compared with standard treatment based on clinical judgment and non-invasive monitoring.

## Materials and methods

### Study design and setting

This was a prospective, parallel, randomised, single-blind study with an allocation ratio of 1:1. The randomised allocation was concealed from the patient only. The treating clinician and outcome assessors were aware of the allocation. From March 2019 to August 2023, all mechanically ventilated patients admitted alive after a non-traumatic cardiac arrest to the emergency department (ED) of Saint-Pierre University Hospital in Brussels, Belgium, were screened for inclusion. The ED is staffed 24/7 by emergency physicians and emergency nurses running the initial assessment and stabilization of OHCA patients before intensive care (ICU) admission.

### Ethics and informed consent

The Saint-Pierre University Hospital ethics boards approved the study design (CE/18–09-12). Consent was waived for initial randomization and treatment; however, written informed consent was required from survivors with preserved neurological function. For all other patients, written consent was obtained from the next of kin. In cases where the patient was unable to provide consent and there was no next of kin, consent was granted by a neutral physician not involved in the study.

### Eligibility and exclusion criteria

Adult, mechanically ventilated patients, with at least 6 ml/Kg of tidal volume, admitted after non-traumatic OHCA with signs of tissue hypoperfusion were eligible for enrolment. Tissue hypoperfusion was defined as an arterial lactate at admission > 4 mmol/L, MAP < 65 mmHg or a capillary refill time (CRT) above 5 s at any moment during ED care. The exclusion criteria were traumatic cardiac arrest, age < 18 years, pregnancy, atrial fibrillation, and prisoner status. Study device malfunction was added as an exclusion criteria after trial registration as some patients could not be randomized as the PVI device connecting cables or power cables were missing or broken in 5 occasions.

### Randomisation

Patients were randomly assigned to the intervention or control group as soon as possible after hospital admission by the treating physician using sealed sequentially numbered envelopes. To allocate participants, a simple computer-generated list without blocks of random numbers was used. The allocation sequence was generated by a research nurse not involved in patient recruitment.

### Control and intervention treatments

All patients were monitored via continuous electrocardiography, non-invasive arterial blood pressure, pulse oximetry and respiratory frequence monitoring. Most patients underwent continuous non-invasive end-tidal carbon dioxide (EtCO_2_) monitoring. Additionally, Radical-97 Pulse CO-Oximeters (Masimo) were used to continuously and noninvasively measure PVi.

**Fig. 2:**
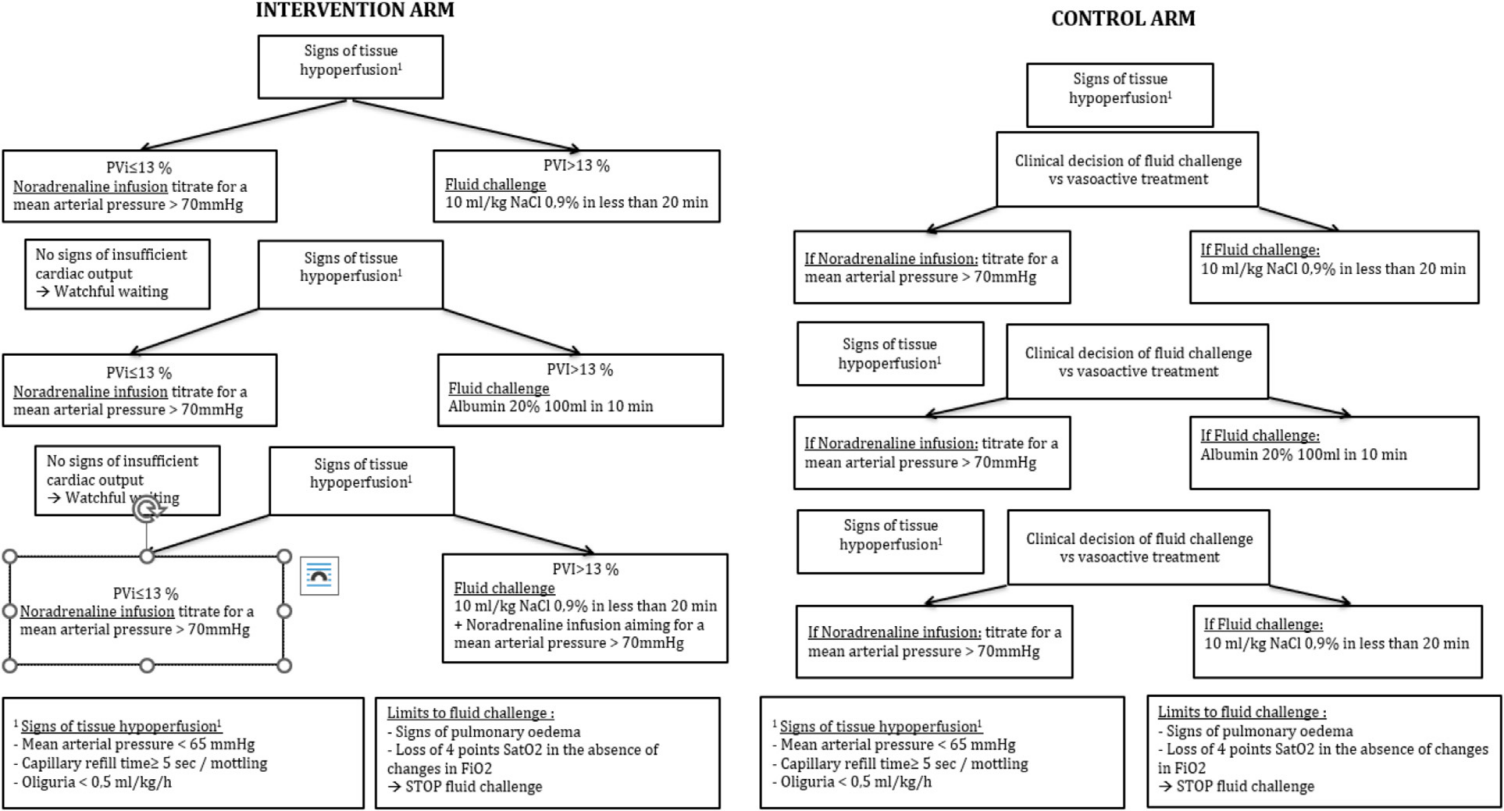
Study protocol. Fig. 2 presents the study intervention. Hemodynamic interventions were titrated in the two study arms to target a mean arterial pressure above 70 mmHg. The choice between fluid challenges and vasopressor therapy was left tot clinical judgement in the control arm. In the intervention arm the choice between fluid challenges and vasopressor therapy was regulated according to PVi values.

Fluid administration and vasopressor treatment was titrated according to the study protocol (NCT03841708, Fig. 2 and Supplementary Data 2). PVi values were accessible only to physicians caring for patient in the intervention group. A study nurse or doctor not involved in patient care were always present during the intervention to check for protocol adherence.

The protocol prescribes decisions on cardiovascular resuscitation (fluid challenge versus vasopressors) based on PVi values in the intervention group, targeting a CRT below 3 s, a MAP above 70 mmHg and a urinary output above 0,5 ml/kg/h. For the control group, cardiovascular resuscitation was based on the clinical judgment of the clinician in charge of the patient, aiming for the same targets of the intervention group. All patients, had to be passive to the ventilator with absence of respiratory efforts. If this could not be achieved with sedation alone, a non-depolarising curare was administered. The intervention was continued until admission to the ICU. Post-resuscitation management was left to the discretion of the treating physician.

### Data collection

The Radical-97 Pulse CO-Oximeter continuously stored waveform SpO_2_ and ORI values at 0.5 Hz. PVi and SpO_2_ data were downloaded, annotated, and analysed according to the statistical analysis methods. OHCA data and demographic information were retrieved from patient records and the Belgian Cardiac Arrest Registry. Arterial blood gas analysis was performed immediately upon hospital admission and at prespecified timepoints on a ABL90 FLEX PLUS blood gas analyser (Radiometer). Cerebral performance category (CPC) and Rankin scale values were retrieved from the Belgian Cardiac Arrest Registry.

### Outcomes

The primary endpoint was lactate clearance assessed at 3 h after the first lactate measurement. Lactate clearance was defined as the difference from baseline (first recorded lactate at admission) to the time point of interest divided by the baseline and multiplied by 100. In patients dead at the moment of lactate clearance calculation, a lactate clearance of 0 was imputed. Other secondary outcomes were lactate clearance at 6, 12 and 24 h, time to reach a lactate < 2 mmol/L, fluid balance at 24 and 48 h after hospital admission, mortality at 24 and 72 h, Sequential Organ Failure Assessment (SOFA) score at 24 h and good neurological outcome at 30 days defined as CPC 1 or 2. Patient surviving less than 24 h were attributed a SOFA score of 24.

### Statistical analyses

Patients were assessed on an intention-to-treat basis.

For hypothesis testing, statistical power calculations could only be performed based on an unpublished small pilot study performed at our institution. For 90% power, a sample size of 40 patients per group was required to detect a 15% relative increase (i.e., 76.9%±16 vs 88.4%) in the lactate clearance at 3 h with a two-sided α-level of 5%. We considered a 15% absolute difference in the lactate clearance as clinically significant given the Cohen’s delta 0.72 indicating a medium effect.

The distribution of continuous data was assessed using the Shapiro–Wilk test. Binary data are presented as counts and percentages. Continuous data are presented as means with standard deviations (SD) or medians with the first and third quartiles (IQR), depending on the distribution of the data. Differences in continuous outcomes between groups are presented as mean differences with 95% confidence intervals (CIs). Two-sided *p* values, obtained using the Mann–Whitney *U* test, were reported for the primary and secondary outcomes. *P* values of less than 0.05 were considered significant. Given the potential for type I errors due to multiple comparisons, the findings for the analyses of secondary outcomes should be interpreted as exploratory. For the primary outcome we calculated the average treatment-effect using teffects ra command on Stata. A posthoc analysis to investigate the role of PVi-guided therapy on good neurological outcome was realized using a logistic regression including age, low-flow duration, lactate at admission and first rhythm during cardiac arrest. All analyses were performed using Stata software version 16 (StataCorp, College Station, TX, USA). The dataset is available upon reasonable request.

## Results

**Fig. 3:**
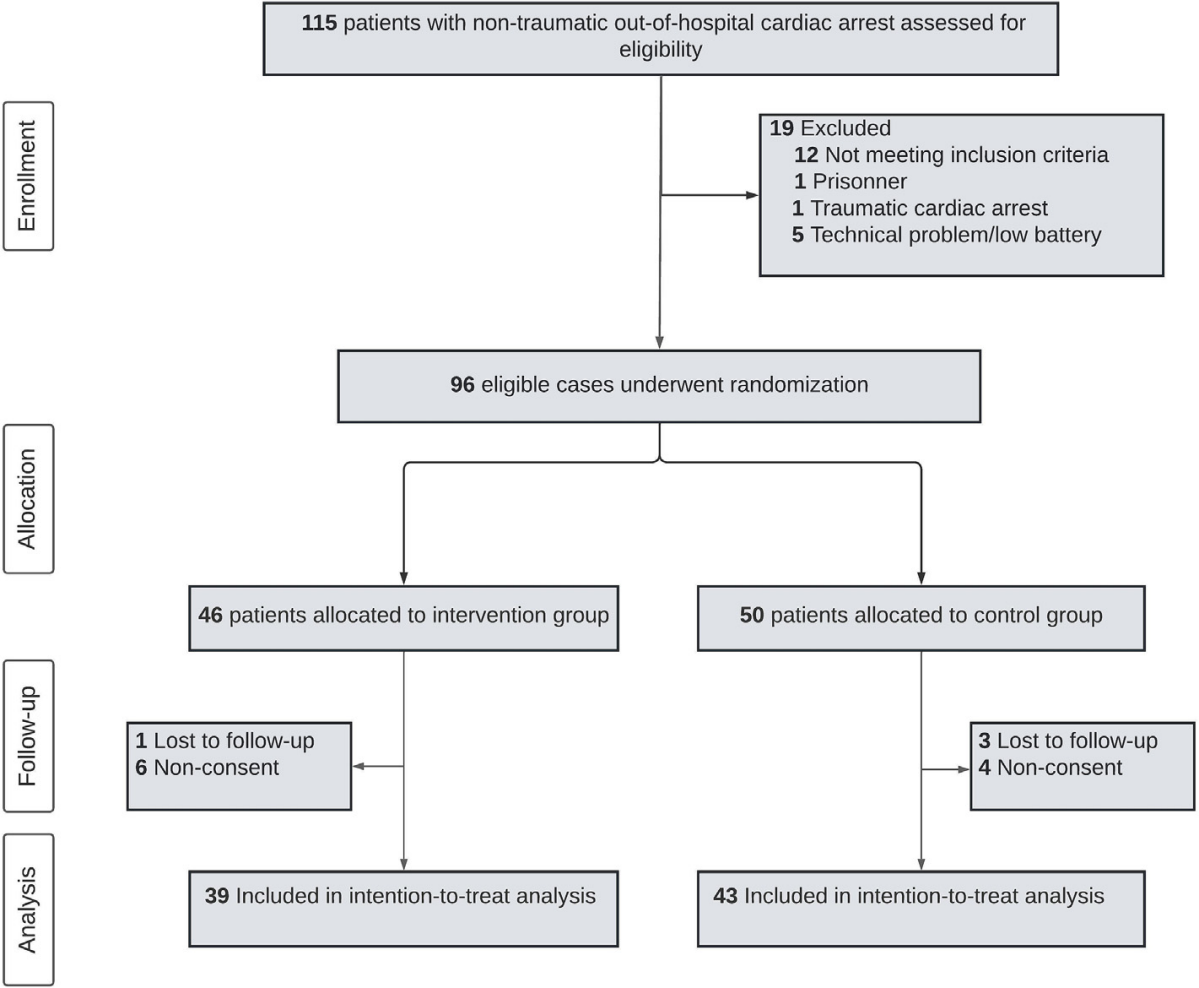
Inclusion flowchart. A total of 115 patients were assessed for eligibility. Of these, 19 patients were deemed ineligible, either due to non-compliance with inclusion criteria or meeting exclusion criteria. 5 patients could not be randomised due to technical problems. Patients were randomized in the emergency department whenever they fulfilled the inclusion criteria. Following the initial allocation, 3 patients in the intervention group and 3 patients in the control group were excluded due to a lack of written consent obtained from either the patient or an authorized next of kin. Upon the completion of the planned recruitment phase, before data analysis, 3 patients from the intervention group and 5 patients from the control group were excluded owing loss in follow-up. This exclusions let to a final analysis cohort of 39 patients in the intervention and 42 in the control group.

Fig. 3 presents a flow diagram outlining patient enrolment. Between March 2019 and August 2023, 115 patients with ROSC following OHCA were admitted to the ED of our study centre. Out of these, 96 patients were enrolled in the trial and underwent randomization (Fig. 3). After the exclusion of 10 patients because of lack of consent, and 4 patients because of loss in follow up the study was concluded after enrolling 82 patients available for the primary analysis.

The baseline demographic, clinical, and CA characteristics of eligible patients are shown in Table 1.

**Table 1:** Demographic and clinical characteristics of the 82 included patients.

		**Clinical-guided hemodynamic therapy**	**PVi-guided hemodyniamic therapy**
*Number of observations*		43	39
**Age, mean (SD), y**		63.4 (17)	58.9 (14.3)
**Female, No. (%)**		13 (30.2)	9 (23.1)
**Height, mean (SD), cm**		170.6 (9.7)	173.1 (7.9)
**Weight, median (IQR), kg**		76.5 (65–90)	75 (64–90)
**mRS, median (IQR)**		1 (0–3)	0 (0–2)
**Arrest Location, No. (%)**			
	**Private home**	24 (58.5)	20 (51.3)
	**Retirement home**	3 (7.3)	3 (7.7)
	**Public space**	12 (29.3)	11 (28.1)
	**Work**	1 (2.4)	3 (7.7)
	**Other**	1 (2.4)	2 (5.12)
**Witnessed arrest, No. (%)**		38 (88.4)	28 (71.8)
**Bystander initiated CPR, No. (%)**		25 (58.2)	18 (46.2)
**Phone-assisted CPR, No. (%)**		8 (19.1)	5 (12.8)
**Initial rhythm, No. (%)**			
	**Asystole**	20 (46.5)	16 (42.11)
	**Pulseless electrical activity**	11 (25.6)	12 (31.6)
	**Ventricular tachycardia**	2 (4.7)	1 (2.6)
	**Ventricular fibrillation**	10 (23.3)	9 (23.5)
**No-flow, median (IQR), min**		5 (2 – 7)	6 (2 – 12)
**First EtCO2, mean (SD), mmHg**		47.5 (18.4)	46.3 (19.2)
**Low-flow, median (IQR), min**		16 (11–21)	16 (13–24)

Abbreviations: EtCO2, end-tidal carbon dioxide; IQR, interquartile range; mRS, modified Rankin scale; PVi, Pleth-Variability index; SD, standard deviation

Table 1 describes the patients’ characteristics at study inclusion in terms of Utstein style cardiac arrest metrics.

Mean age (±SD) was 61.2 (±15.9) years, CA was witnessed in 80.4% of cases and shockable rhythm was the presenting rhythm in 27.1% of cases. At admission, median (IQR) lactate was 9 mmol/L (6.2–12.2 mmol/L) and CRT was 4.3 s (±1.75 s) (Table 2).

**Table 2:** Clinical characteristics of the 82 patients at ED admission.

	**Clinical-guided hemodynamic therapy**	**PVi-guided hemodyniamic therapy**
*Number of observations*	43	39
**Lactate at admission, median (IQR), mmol/L**	8.7 (6.1–12.2)	9.4 (6.2–12.5)
**Heart rate, mean (SD), bpm**	88 (25.4)	90.6 (23)
**Mean arterial pressure, median (IQR), mmHg**	79 (69–94)	79 (63–95)
**Respiratory frequency, mean (SD), Bpm**	15.9 (3.7)	16.2 (3.2)
**PVi, median (IQR)**	19 (11–31)	17 (10–24)
**Tidal volume, median (IQR), mL/kg**	7.32 (6.5–8)	7.1 (6.7–7.6)
**PEEP, median (IQR), cm H_2_0**	5 (5–6)	5 (5–5)
**Capillary refill time, mean (SD) seconds**	4.4 (1.7)	4.5 (2)

Abbreviations: bpm, beat per minute; Bpm, Breath per minute; IQR, interquartile range; ml/kg, milliliters per kilogram; mmHg, millimiters of mercury; PEEP, positive end expiratory presssure; PVi, pleth variability index; SD, standard deviation

Table 2 present clinical characteristics and ventilatory conditions of study patients at hospital admission

### Hemodynamic management during ED admission

During the ED stay, the median duration of PVi monitoring from hospital admission until ICU admission was 119 min (80–188 min). Isotonic fluid administration was not different between the control and intervention groups (675 ml [300–1000 ml] versus 500 [200–820 ml]; *p* = 0.21) (Table 3).

**Table 3:** Interventions during study ED study period.

	**Clinical judgment-guided hemodynamic therapy**	**PVi-guided hemodyniamic therapy**	**p value**
*Number of observations*	43	39	
**Total fluids, median (IQR), ml**	675 (300–1000)	500 (200–820)	0.21
**Albumin, No. (%)**	13 (30.2)	8 (20.5)	0.31
**Mean PVi, median (IQR), %**	17 (11–27)	14 (10–17)	0.11
**Norepinephrine, No. (%)**	35 (81.4)	29 (74.4)	0.44
**Norepinephrine at ED discharge, median (IQR), µcg/kg/min**	0.8 (0–2.5)	0.4 (0–1.4)	0.17
**Diuresis, median (IQR), ml/kg/h**	0.14 (0–0.79)	0.34 (0.06–0.76)	0.46
**PVi monitoring time, median (IQR), minutes**	119.1 (80–188)	118.5 (83–175)	0.76

Abbreviations: ED, emergency department; IQR, interquartile range; No., number; PVi, Pleth-Variability index; SD, standard deviation; µcg/kg/min, microgramm/kilogramm/minute.

Table 3 shows the interventions during the study intervention time in the emergency department.

Albumin was administered in 30.2% of patients in the control group versus 20.5% in the PVi-guided group (*p* = 0.31). Noradrenaline infusion was initiated in 81.4% of patients in the control group versus 74.4% of patients in the intervention group (*p* = 0.44). The noradrenaline dose at ED discharge was not different in the two groups (0.8 µcg/kg/min [0–2.5 µcg/kg/min] versus 0.4 µcg/kg/min [0–1.4 µcg/kg/min]; *p* = 0.17).

### Lactate clearance

The primary outcome, median lactate clearance at 3 h, was 57.4% (IQR: 27.7–75.8%) and 61.5% (IQR: 39.3–74.7%) in the control and intervention groups respectively (Table 4), with a mean difference of 4.9% (95% CI, −7.5–17.2; *p* = 0.44) between the two groups. No differences in lactate clearance were observed at 6 (69.1% [IQR: 46.1–82.4] versus 71.1 [IQR: 45.5–82.2]; *p* = 0.70), at 12 (70.8% [IQR: 1.2–85.4%] versus 80.5% [IQR: 62.5–90%]; *p* = 0.13) or at 24 h (81.1% [17.8–87.8%] versus 86.1% [64.5–90.4%]; *p* = 0.34). Time to achieve a lactate below 2 mmol/L was 13.7 h (IQR: 5.9–48) and 7.4 h (IQR: 4.6–25.1) in the control and intervention groups respectively (*p* = 0.17).

**Table 4:** Outcomes Collected During follow up.

		**Clinical-guided hemodynamic therapy**	**PVi-guided hemodyniamic therapy**	**p value**
	*Number of observations*	43	39	
**Primary outcome**				
Lactate clearance at 3 h, median (IQR), %		58.2 (36.7–77.8)	63.7 (41.6–77.6)	0.55
**Secondary outcomes**				
Lactate clearance at 6 h, median (IQR), %		69.1 (46.1–82.4)	71.1 (45.5–82.4)	0.62
Lactate clearance at 12 h, median (IQR), %		70.8 (−6.6–85.4)	80.5 (62.5–90)	0.11
Lactate clearance at 24 h, median (IQR), %		81.1 (17.8–87.8)	86.1 (64.5–90.4)	0.4
Time to lactate < 2 mmol/L, median (IQR), hours		13.7 (5.9–48)	12.3 (5.1–30.3)	0.24
Fluid balance at 24 h, median (IQR), ml		130 (0–1592)	36 (−584–1068)	0.28
Fluid balance at 48 h, median (IQR), ml		284 (−1102–1844)	−294 (−1368–2110)	0.84
SOFA score at 24 h, median (IQR)		13 (10–24)	10 (8–24)	0.03
CPC 1–2 at hospital discharge, No. (%)		8 (18.6)	11 (28.2)	0.3
Mortality at 24 h, No. (%)		12 (27.9)	6 (15.4)	0.17
Mortality at 72 h, No. (%)		18 (41.9)	15 (35.5)	0.75

**Other outcomes measured**				
Lactate at 3 h, median (IQR), mmol/L		2.8 (2–5.4)	2.7 (1.7–7.1)	0.82
Lactate at 6 h, median (IQR), mmol/L		2.4 (1.5–3.8)	1.8 (0.8–5.2)	0.33
Lactate at 12 h, median (IQR), mmol/L		1.8 (1.2–3.2)	1.4 (0.9–2.6)	0.25
Lactate at 24 h, median (IQR), mmol/L		1.2 (0.8–1.5)	1.1 (0.8–1.7)	0.5
Rearrest at ED, No. (%)		9 (20.9)	4 (10.3)	0.18
mRS at hospital discharge, median (IQR)		6 (5–6)	6 (3–6)	0.31

Abbreviations: CPC, cerebral performance category ED, emergency department; IQR, interquartile range; No., number; mRS, modified Rankin Scale; SD, standard deviation; SOFA, sequential organ failure assessment.

Table 4 presents crude lactate values as well study primary and secondary outcomes collected during the follow up in the emergency department and intensive care unit. The primary outcome was lactate clearance. Secondary outcomes were fluid balance at 24 and 48 h, sequential organ failure assessment score (SOFA) at 24 h, good neurological outcome defined as cerebral performance status 1 or 2 at hospital discharge. SOFA score was lower in the intervention group than in control group. No other significant differences were observed.

### Fluid balance and SOFA score

Fluid balance was calculated in patients surviving 24 h or more. Thirty-one patients in the control and thirty-three in the intervention groups were alive at 24 h post-ROSC (*p* = 0.17). Fluid balance was 130 ml (0–1592 ml) in the control group and 36 ml (−584–1068 ml) in the intervention group (Table 4) (*p* = 0.28). No differences in fluid balance were observed at 48 h between the control and intervention group (456 ± 2694 versus 328 ± 2417 mL, *p* = 0.84). The SOFA score was higher at 24 h in the control group (13^10^, ^11^, ^12^, ^13^, ^14^, ^15^, ^16^, ^17^, ^18^, ^19^, ^20^, ^21^, ^22^, ^23^, ^24^ versus 10^8^, ^9^, ^10^, ^11^, ^12^, ^13^, ^14^, ^15^; *p* = 0.04).

### Good neurological survival at hospital discharge

Favourable neurological outcome at hospital discharge was not statistically superior in the intervention group (18.6% vs 28.2%; *p* = 0.3).

A logistic model to analize the role of PVi-guided therapy on survival with CPC 1–2 showed no statistically significant role for PVi-guided hamodynamic therapy (Supplementary Data 3).

## Discussion

In this randomised trial, we compared a PVi-guided hemodynamic protocol to a protocol based on clinical judgment. We found no significant differences in the cardiovascular resuscitation provided nor in the outcomes of the two strategies in terms of lactate clearance. The findings do not support the use of the PVi to guide fluid administration and vasopressor treatment in OHCA patients admitted to the ED.

Early in-hospital hemodynamic optimisation of patients with signs of hypoperfusion, as per guidelines, initially relies solely on fluid challenges^9^ although fluid responsiveness is present in only half of hemodynamically unstable patients.^20^ Fluid administration to avoid hypotension, which is associated with worse neurological outcome,^3^, ^6^ may be ineffective in some^20^ patients and could expose them to the risks of fluid intolerance such as pulmonary oedema and systemic venous congestion.^21^ Treating hypotension using norepinephrine is a viable alternative and higher norepinephrine doses have been associated with reduced myocardial injury.^7^

Tailored cardiovascular resuscitation with individualized fluid and vasopressor titration in OHCA patients with inadequate tissue perfusion during the early hospital phase is therefore challenging.^22^ Correctly assessing fluid responsiveness in the early phases of hospital admission is difficult. Transthoracic echocardiography is a non-invasive and accessible technique to assess shock^23^ but is time-intensive and difficult to perform in the ED. Moreover, a reliable assessment of fluid responsiveness requires either passive synchronization of the patient with invasive mechanical ventilation or a passive leg raising manoeuvre, which is difficult to implement early post-arrest in the ED.^23^ Other dynamic indices of fluid responsiveness such as SVV^24^ or PPV^25^ require invasive measures from an arterial catheter which is not available in the early post-arrest phases. Conversely, PVi can continuously and noninvasively predict fluid responsiveness in critical patients under mechanical ventilation, provided the absence of respiratory efforts and a sinus rhythm.^17^

We observed a higher lactate at admission (9 mmol/L [6.2–12.2 mmol/L]) than previously reported,^26^ probably due to selection bias from recruiting hemodynamically unstable patients and potentially to shorter scene-to-hospital transport times.

Besides admission lactate, the findings of our study align with those of previous studies reporting that early hemodynamic assessment and treatment are challenging, with frequent episodes of hypotension and difficulties in rapidly achieving hemodynamic objectives.^27^ A previous hospital study reported a 33% incidence of rearrests, and reported that only 76% of patients achieved a MAP above 65 mmHg and that only 83% of the patients had a decreasing lactate in 24 h.^27^ In comparison, we observed a lower rearrest proportion and a tendency towards lower rearrests in the intervention group (10% versus 20%; *p* = 0.18).

Although studies repeatedly reported that faster lactate clearance and higher blood pressures are associated with better outcomes the clinical implementation of these findings remains unclear. A recent randomised, multicentre study involving OHCA patients after admission to the ICU, compared the effect of the implementation of two blood pressure targets, 63 and 77 mmHg.^8^ No difference between the two groups was observed on the primary composite endpoint of death from any cause or hospital discharge with a CPC of 3 or 4 within 90 days. It is important to note that in this study, interventions to achieve blood pressure targets were initiated only after ICU admission and therefore do not reflect potential benefits or harms of specific MAP targets early after ROSC.^10^ In contrast, our study intervention lasted the duration of the ED stay when its potential benefit was higher despite its limited duration.

Lactate clearance, the primary outcome, was specified and designed a priori in order to measure a reproducible microcirculatory index repeatedly associated with the outcome of cardiac arrest patients.^26^, ^28^ In our study, additional monitoring of PVi did not reduce the lactate clearance during the first 24 h. A possible explanation for this is the impracticability of reliably measuring PVi in vasoconstricted and hemodynamically unstable patients during the early phases of in-hospital management after resuscitation following OHCA.^29^ Moreover, extreme vasoconstriction from adrenaline use during resuscitation, motion artefacts associated with frequent patient and stretcher movement and cold extremities may further decrease the reliability of PVi in this setting.^30^

SOFA was lower in patients in the PVi-guided group. SOFA was a secondary outcome for which the study was not powered. The multiple comparisons and the paucity of differences in the ED care of the two groups suggest that this result should be considered cautiously as hypothesis-generating. Neurologically intact survival with CPC 1–2 was observed in 28.8% and 18.6% of patients in the intervention and control group respectively. A tendency towards increased neurologically intact survival was present in patients from the PVi-guided protocol. Adequately powered studies based on this observation are needed to test whether a PVi-based protocol at ED admission could be associated with neurological intact survival. It is worth noting that neurological intact survival was lower in our study than reported in the literature.^26^ This may be explained by selection bias, as we recruited hemodynamically unstable patients, and survival bias, which is inherent in studies enrolling patients later in their care process.^8^ After adjusting for variables associated with neurological intact survival PVi-guided therapy was not associated with CPC 1–2 (OR 2.00 [0.47 – 8.53]; *p* = 0.35 (Supplementary Data 3).

Overall, contrary to our initial hypothesis, PVi does not seem instructive for early in-hospital management of OHCA although it was associated with a reduced SOFA at 24 h and a tendency towards lower re-arrest during ED stay.

This study has several limitations. First, left and right ventricular dysfunction may have resulted in falsely elevated PVi that were not associated with fluid responsiveness. Moreover, adrenaline and noradrenaline use could have hindered PVi predictive capacity. Second, echocardiography was not systematically assessed in all patients. Third, the ED personnel involved in the study were not blinded to the treatment allocation. Fourth, the training for PVi-guided hemodynamic management might have been insufficient given the complexity of the required task in a stressful environment. Finally, the generalisability of the study is low as recruitment was performed by a single centre in a specific tertiary care setting. However, the randomised design with balanced groups remains a strength of our study.

## Conclusion

A PVi-guided hemodynamic optimisation strategy in patients admitted to the ED with signs of hypoperfusion did not improve lactate clearance compared with clinical judgment alone. For early in-hospital settings, the findings do not support the use of an PVi-based approach after resuscitation from OHCA to maximise lactate clearance.

## Data sharing

Data are available from the corresponding author upon reasonable request.

## Conflicts of interest

Stefano Malinverni received material support for another study from Masimo Corporation.

All the other authors stated that they have no conflict of interest

## Funding source declaration

This work was supported by the King Baudouin Foundation (Belgium) and the Fund Désiré and Simone Drieghe Miller (Belgium). The funding sources had no role in the study design, collection, analysis, and interpretation of data, the writing of the report, or the decision to submit the article for publication.

## CRediT authorship contribution statement

**Stefano Malinverni:** Writing – review & editing, Writing – original draft, Validation, Supervision, Project administration, Methodology, Investigation, Funding acquisition, Formal analysis, Data curation, Conceptualization. **Paul Dumay:** Writing – original draft, Validation, Project administration, Investigation. **Pierre Domont:** Writing – original draft, Validation, Investigation. **Marc Claus:** Writing – original draft, Validation, Project administration, Investigation. **Antoine Herpain:** Writing – original draft, Validation, Project administration, Investigation. **Jolan Grignard:** Writing – original draft, Validation, Project administration, Investigation. **Silvia Matta:** Writing – original draft, Validation, Project administration, Investigation. **Fatima Zohra Bouazza:** Writing – original draft, Validation, Project administration, Investigation. **Queitan Ochogavia:** Writing – original draft, Validation, Project administration, Investigation.

## Declaration of competing interest

The authors declare that they have no known competing financial interests or personal relationships that could have appeared to influence the work reported in this paper.
