# Supplementary material for: Postresuscitation pleth variability index-guided hemodynamic management of out-of-hospital cardiac arrest survivors: A randomised controlled trial
Source: Resusc Plus. 2025 Mar 19;23:100933. doi: 10.1016/j.resplu.2025.100933 (PMC11995752; doi:10.1016/j.resplu.2025.100933)
Supplement: Supplementary Data 2 [file mmc2.pdf]

Supplementary materials 1

PVi guided hemodynamic management protocol

Every patient was sedated with titrated midazolam or propofol and remifentanil.

If patient – ventilator asynchronies persisted in presence of an adequate sedation, muscle paralysis with atracurium 0.4-0.5 mg/kg IV was recommended.

Observational arm

In the Observational (control) arm, in case of inadequate tissue perfusion (CRT≥5 sec, mean arterial pressure < 65 mmHg, lactate at admission > 4 mmol/L), the therapeutical decision of a fluid challenge versus the use of noradrenaline was left to the clinician.

The clinician was blinded to PVi values.

If the clinician opted for a fluid challenge:

- The first fluid challenge had to be 10 ml/Kg of NaCl 0.9%
- The second fluid challenge had to be 100 ml of Albumin 20%
- The third and all the following fluid challenges had to be 10 ml/Kg of NaCl 0.9%

If the clinician opted for a vasoactive treatment with titrated noradrenaline was recommended with a mean arterial pressure target of 70 mmHg.

Safety limits: Fluid challenge was interrupted if the patient lost >4% points of $SatO_2$ or if pulmonary oedema signs appeared during the fluid challenge

Interventional arm

In the interventional (PVi guided) arm, in case of inadequate tissue perfusion (CRT≥5 sec, mean arterial pressure < 65 mmHg, lactate at admission > 4 mmol/L), the therapeutical decision of a fluid challenge versus the use of noradrenaline was guided by PVi values.

If PVi was superior to 13% a fluid challenge was recommended.

In case of a fluid challenge:

- The first fluid challenge had to be 10 ml/Kg of NaCl 0.9%
- The second fluid challenge had to be 100 ml of Albumin 20%
- The third and all the following fluid challenges had to be 10 ml/Kg of NaCl 0.9%

If PVi was inferior or equal to 13% vasoactive treatment with titrated noradrenaline was recommended with a mean arterial pressure target of 70 mmHg.

Safety limits: Fluid challenge was interrupted if the patient lost >4% points of $SatO_2$ or if pulmonary oedema signs appeared during the fluid challenge
