# Supplementary material for: Postresuscitation pleth variability index-guided hemodynamic management of out-of-hospital cardiac arrest survivors: A randomised controlled trial
Source: Resusc Plus. 2025 Mar 19;23:100933. doi: 10.1016/j.resplu.2025.100933 (PMC11995752; doi:10.1016/j.resplu.2025.100933)
Supplement: Supplementary Data 3 [file mmc3.pdf]

Supplementary Table 1:

Logistical multivariable regresssion adjusted for factors associated with mortality

Odds ratio for neurological intact survival defined as CPC 1-2

	**Odds Ratio (95% CI)**	**p value**
PVi guided therapy	2.00 (0.47 to 8.53)	0.350
Shockable initial rhythm	7.52 (1.27 – 44.45)	0.026
Age (years)	0.93 (0.88 to 0.98)	0.006
Lactate at admission (mmol/L)	0.85 (0.72 to 1.00)	0.062
Low-flow time (min)	0.90 (0.82 - 0.99)	0.025

Abbreviations: CI, Confidence Interval; Pvi, pleth variability index

.
